# Age-specific trend and birth cohort effect on different histologic types of uterine corpus cancers

**DOI:** 10.1038/s41598-022-21669-4

**Published:** 2023-01-19

**Authors:** Yi-Jou Tai, Chun-Ju Chiang, Ying-Cheng Chiang, Chia-Ying Wu, Wen-Chung Lee, Wen-Fang Cheng

**Affiliations:** 1 Department of Obstetrics and Gynecology, College of Medicine, National Taiwan University, Taipei, Taiwan, ROC; 2 Graduate Institute of Clinical Medicine, College of Medicine, National Taiwan University, Taipei, Taiwan, ROC; 3 Institute of Epidemiology and Preventive Medicine, College of Public Health, National Taiwan University, Taipei, Taiwan, ROC; 4 Taiwan Cancer Registry, Taipei, Taiwan, ROC; 5 Department of Obstetrics and Gynecology, Ministry of Health and Welfare Nantou Hospital, Nantou City, Taiwan, ROC; 6 Graduate Institute of Oncology, College of Medicine, National Taiwan University, Taipei, Taiwan, ROC

**Keywords:** Cancer epidemiology, Endometrial cancer

## Abstract

To evaluate the uterine corpus cancer incidence rates, age-specific trends, and birth cohort patterns by different histologic types. We conducted a retrospective cohort study of uterine cancer patients (n = 28,769) of all ages from the National Cancer Registry of Taiwan between 1998 and 2017. We estimated the incidence trends, average annual percent changes (AAPCs), and cancer-specific survival (CSS) rate for the two main subtypes (endometrioid and nonendometrioid) of uterine cancer in Taiwan. During the study period, uterine corpus cancer incidence rates increased over time from 5.3 to 15.21 per 100,000 women. Incidence trends for endometrioid carcinoma increased in all age groups (positive AAPCs > 5% for each age group), and the rise was steeper among women aged 50 years and younger. For nonendometrioid carcinomas, incidence rates increased among women over 50 years. The CSS rate improved among women with stage I (hazard ratio [HR] 0.63, 95% confidence interval [CI] 0.49–0.81) and stage III (HR 0.72, 95% CI 0.58–0.90) endometrioid carcinomas after 2013 compared with those during 2009–2012. However, the CSS rate remained unchanged for nonendometrioid carcinomas. Age, diagnostic period, stage and histologic types were significant factors associated with the 5-year CSS rate. We found that the incidences of both endometrioid and nonendometrioid carcinomas continued to increase among contemporary birth cohorts. Etiologic research is needed to explain the causes of these trends.

## Introduction

Uterine corpus (hereafter referred to as uterine) cancer is the most common gynecologic malignancy in the U.S. and other Western countries^1^. The incidence of uterine cancer has increased in both consecutive generations and over time. The largest increases were observed in South Africa and several Asian countries^2^. In Taiwan, in 2010, uterine cancer surpassed cervical cancer as the most common gynecologic malignancy, and in 2017, approximately 2700 women were newly diagnosed^3^. In addition, the incidence of uterine cancer doubled in the last decade, and it increased by approximately fourfold over a 20-year period with a continuously rising trend^4,5^. The rise in the incidence of uterine cancer was attributable to the rise in obesity, based on the association between excess body weight and uterine cancer shown in epidemiological cohort studies^6,7^. The prevalence of obesity has increased substantially across all population groups worldwide^8–10^. However, the prevalence of overweight and obesity was modest in Taiwan, and the upward trend in obesity rates has apparently leveled off in recent years^11–13^. Lin et al. reported a remarkable finding that the greatest increases in breast and uterine cancer rates occurred among young Taiwanese women, with a uniquely high rate of breast cancer among younger Taiwanese women^14^. These findings prompted us to investigate the current incidences and evaluate the birth cohort effect on uterine cancers in Taiwan.

More than 90% of uterine cancer is of endometrial origin, and other types of uterine cancer, such as uterine sarcoma, are much less common. Beyond the conventional diagnosis, there are two distinct types of endometrial cancer based on histology and molecular features. In this study, we analyzed and classified endometrial cancer subtypes into endometrioid (type I) and nonendometrioid (type II) carcinomas. Type I endometrial cancer is a typical endometrioid type that accounts for 80–90% of all sporadic endometrial cancers, and these cancers are primarily associated with excess unopposed estrogen exposure. This type includes well to moderately differentiated carcinomas, and the prognosis is good. Type II endometrial cancer accounts for 10–20% of all endometrial cancers, independent of estrogen exposure. They are usually poorly differentiated and nonendometrioid carcinoma including serous and clear cell carcinomas. Common risk factors for endometrioid carcinoma include nulliparity, obesity, delayed menopause, endometrial hyperplasia, polycystic ovarian syndrome, and chronic anovulation^15^. In contrast, the epidemiology and biology of nonendometrioid carcinoma are not well characterized. Similar to endometrioid carcinoma, the incidence of nonendometrioid carcinoma has also increased based on data from the Surveillance, Epidemiology, and End Results (SEER) program database in the US. However, the cause of the increase remains unclear^16^. Age-specific and birth cohort patterns are important etiologic clues but have not been recently examined for nonendometrioid carcinoma. Therefore, it is necessary to identify the epidemiology of nonendometrioid carcinoma.

In the present retrospective population-based study, we investigated the incidences associated with different histologic types of uterine cancer and estimated the annual percent change in trend analysis. In addition, we assessed the effect of different factors on survival.

## Methods

### Study design and data source

We conducted a retrospective cohort study of uterine cancer patients of all ages from the National Cancer Registry of Taiwan between 1998 and 2017. The Taiwan Cancer Registry (TCR) is a nationwide population-based cancer registry established in 1979 by the Ministry of Health and Welfare^17,18^. With the enactment of the Cancer Control Act in 2003, the data quality and completeness (approximately 98%) of the TCR database have been improved and continuously maintained. Thus, the TCR is one of the highest-quality cancer registries in the world^19^. The diagnosis of primary cancer of the uterus was based on the International Classification of Diseases for Oncology, third edition (ICD-O-3: C54.0–C54.3, C54.8–C54.9) and classified by histological codes. The histologic subtypes of uterine carcinomas included endometrioid adenocarcinoma (codes 81403, 82603, 82623, 82633, 83233, 83803, 83823, 83833, 83843, and 85703), nonendometrioid adenocarcinoma (codes 83103, 84413, 84603, 84613, 84,803, 84813, 85603, 89503, 89513, and 89803), uterine sarcoma (codes 87143, 88003, 88023, 88053, 88513, 88903, 88,913, 88963, 89203, 89303, 89313, 89333, 89353, and 90403), and other carcinomas (80003, 80103, 80133, 80203, 80303, 80333, 80413, 80503, 80703, 80713, 80723, 82463, 82553, 83813, 85743, 88013, 89003, 89013, 89633, 91003, 91043, and 91053)^20^. The population data for women in Taiwan between 1998 and 2017 were extracted from the National Household Registration Census Database provided by the Department of Statistics of the Ministry of the Interior. All causes of death in Taiwan were abstracted from the National Death Registration Database provided by the Ministry of Health and Welfare. The Ninth and Tenth Revisions of the International Classification of Diseases (ICD) were used to record the cause of death before and after 2008, respectively. Uterine cancer death (ICD‐9 code: 182; ICD‐10 code: C54) was considered the cancer-specific cause in this study.

Cancer staging information for uterine cancer was collected in the TCR database after 2009. The stages were assigned according to the TNM staging system determined by the American Joint Committee on Cancer^21^. The final stage assignment was based on the best available information on the stage of the disease. Thus, when preoperative therapy was not performed, we used the pathologic stage, and when neoadjuvant therapy was performed first, we used the clinical stage.

### Statistical analyses

The incidence rates of uterine cancers were calculated from 1998 to 2017 for the total, different histological subtypes, and three age groups (< 50, 50–59, ≥ 60 years). All rates were presented per 100 000 women and age-adjusted to the 2000 world standard population for trend analysis to minimize the effect of changes in age distributions over time. Trends in age-specific incidences of uterine cancer were estimated in 5-year age groups for the calendar year (1998–2002, 2003–2007, 2008–2012, 2013–2017), histological subtypes, and birth cohorts (1933–1937 to 1983–1987 for 11 birth cohorts).

To quantify the incidence trends, we first evaluated the annual percent change (APC) with a joinpoint regression analysis (Joinpoint Regression Program, version 4.7, February 2019; National Cancer Institute, Bethesda, MD). Then, we calculated the average APC (AAPC) and confidence intervals (CIs). We calculated AAPCs for the two main subtypes (endometrioid and nonendometrioid) across all ages, for the three age groups (< 50, 50–59, ≥ 60 years), and for each age group. Because there were only 12 patients under the age of 25, the AAPC for each age group was merely represented by those over the age of 25. The best fitting trend lines where the rate changed significantly were used for Monte Carlo permutation tests. The 95% CI of the AAPC excluding zero was considered statistically significant (*P* < 0.05).

All uterine cancer patients with stage data were followed through individual data using their unique personal identity number linked to the Death Registration Database until December 31, 2018. According to cancer stage and two time periods of diagnosis (2009–2012 and 2013–2017), Kaplan‒Meier analysis was used to evaluate the cancer-specific survival (CSS) rate of women with uterine endometrioid and nonendometrioid carcinomas. A multivariable Cox proportional hazards regression model was used to identify covariates that were significantly associated with the CSS rate, including age, diagnostic period, stage, and histological subtypes. Hazard ratios (HRs) and associated 95% confidence intervals (CIs) were calculated in the multivariable models. Two-sided statistical significance was defined as *P* < 0.05. All analyses were performed with SAS software version 9.4 (SAS Institute, Inc., Cary, NC, USA).

### Ethics approval

The Research Ethics Committee of National Taiwan University Hospital approved this study (202007069RINA). Informed consent was waived by National Taiwan University Hospital Research Ethics Committee. This study was conducted in accordance with the Declaration of Helsinki and its later amendments.

## Results

### Age-adjusted uterine cancer incidences over time for various histologic types

**Figure 1:**
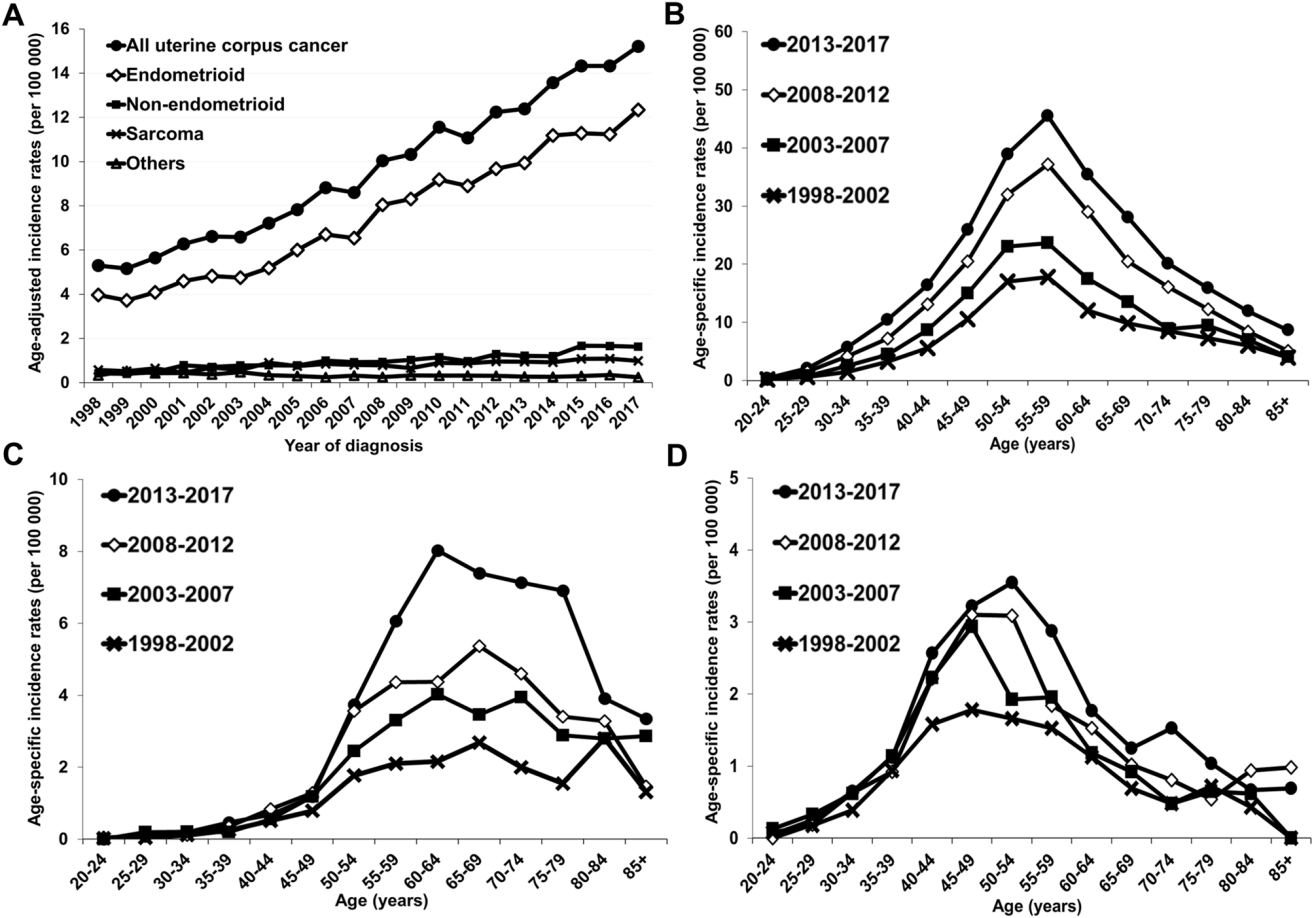
Trends in the incidences of uterine corpus cancer in Taiwan during 1998–2017. (**A**) Age-adjusted incidence of uterine corpus cancers, stratified by histologic type. Age-specific incidence of (**B**) endometrioid carcinoma, (**C**) nonendometrioid carcinoma, and (**D**) uterine sarcoma, grouped by calendar year.

We identified 28,769 women diagnosed with uterine cancer over a 20-year period in Taiwan from 1998 to 2017 (Table 1). Among all uterine cancers, endometrioid carcinoma comprised 72.8–79.7%, nonendometrioid carcinoma comprised 9.4–11.3%, and uterine sarcoma comprised 6.8–10.5%. The median age at diagnosis was older among patients with nonendometrioid carcinoma than among those with endometrioid carcinoma or sarcoma. The age-adjusted incidence rates of all uterine cancers increased threefold from 1998 to 2017 (5.3/100,000 in 1998 and 15.2/100,000 in 2017) (Fig. 1A). A parallel increase in the incidence of endometrioid carcinoma (3.97/100,000 in 1998, 12.3/100,000 in 2017) accounted for the overall upward trend in all uterine cancers. Notably, the age-adjusted incidence rates of nonendometrioid carcinoma showed a fourfold increase (0.42/100,000 in 1998 to 1.63/100,000 in 2017). In contrast, although the incidence of uterine sarcoma also increased, it remained relatively low (0.58/100,000 in 1998 and 0.99/100,000 in 2017).

**Table 1:** Distribution of histologic types and age at diagnosis for 28,769 women with uterine corpus cancer from 1998 to 2017 in Taiwan.

Histologic type	Year of diagnosis			
	1998–2002 (n = 3229)	2003–2007 (n = 5154)	2008–2012 (n = 8434)	2013–2017 (n = 11,952)
**Cases (%)**				
Endometrioid	2350 (72.8)	3859 (74.9)	6733 (79.8)	9521 (79.7)
Nonendometrioid	305 (9.4)	552 (10.7)	831 (9.9)	1356 (11.3)
Sarcoma	339 (10.5)	526 (10.2)	635 (7.5)	816 (6.8)
Others	235 (7.3)	217 (4.2)	235 (2.8)	259 (2.2)
**Age at diagnosis, median (range, years)**				
Endometrioid	52 (22–90)	52 (18–93)	54 (21–94)	55 (19–96)
Nonendometrioid	57 (24–90)	59 (25–93)	58 (24–96)	61 (27–93)
Sarcoma	46 (22–80)	46 (16–84)	49 (18–87)	51 (17–90)
Others	52 (19–90)	52 (19–94)	55 (14–97)	60 (13–102)

We depicted the age-specific incidence rates of uterine cancers during the four different time periods. The peak incidences of endometrioid carcinoma consistently occurred in the 55- to 59-year age group, but the slopes became steeper between 1998 and 2002 and between 2013 and 2017 (Fig. 1B). The age-specific incidence rates of nonendometrioid carcinoma also increased in all time periods (Fig. 1C). In contrast to the narrow, bell-shaped pattern observed for the incidences of endometrioid carcinoma, the peak incidences of nonendometrioid carcinoma varied between the 60- and 80-year age groups in the different time periods. The incidence of nonendometrioid carcinomas increased, and the steepest slope also occurred in the most recent period (2013–2017). The age-specific incidence rate of uterine sarcoma increased, and the peaks occurred at ages 45–54 in all four time periods (Fig. 1D). Again, the steepest slope was observed from 2013 to 2017.

**Figure 2:**
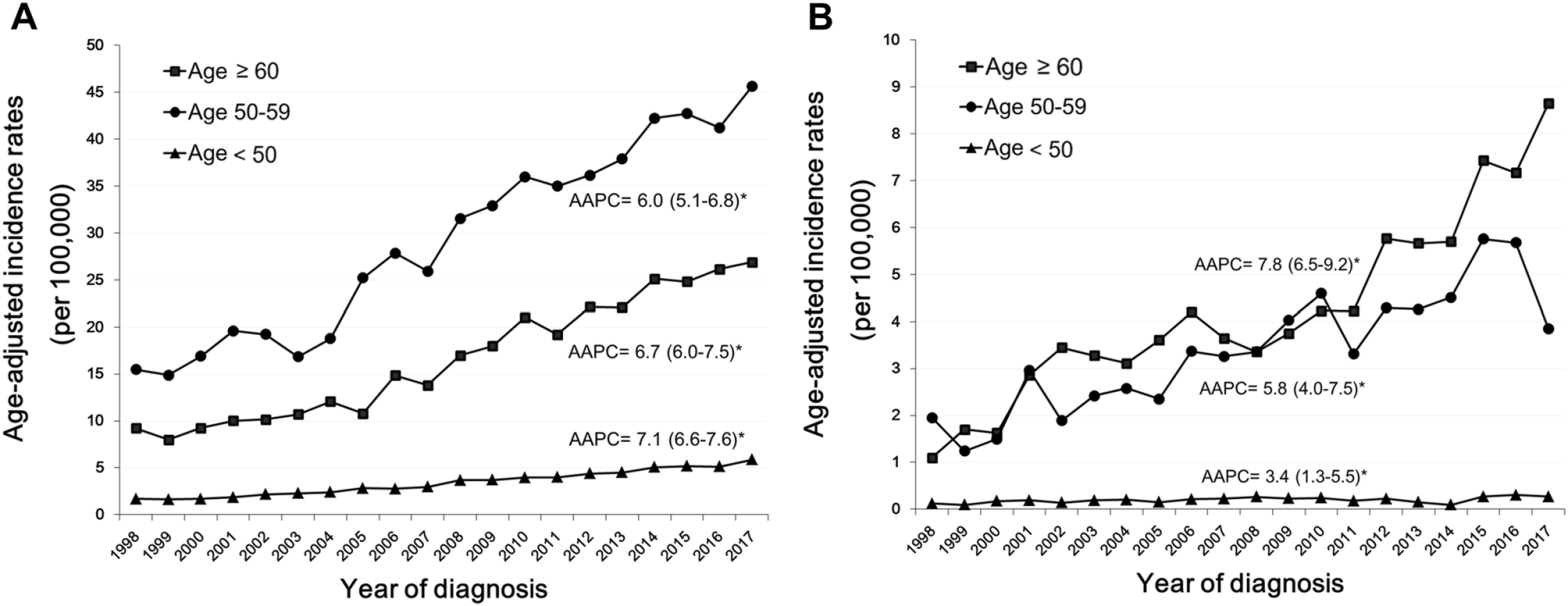
Incidence rates of endometrial cancer in Taiwan between 1998 and 2017. Age-adjusted incidences of (**A**) endometrioid and (**B**) nonendometrioid carcinoma in the 3 age groups. Trends are summarized by the annual percentage change estimate (95% CI). (*) Significantly different than zero at *P* < 0.05.

We used AAPCs to quantify temporal trends of endometrioid and nonendometrioid carcinomas for patients of different age groups from 1998 to 2017 (Supplementary Figs. 1, 2). The incidence of endometrioid carcinoma always showed positive AAPCs > 5% for each age group, whereas the incidence of nonendometrioid carcinoma showed significant increases in AAPCs among women aged 50 and older. The incidence rates for endometrial cancer in Taiwan were highest among postmenopausal women. We further evaluated the age-adjusted incidences of both endometrioid and nonendometrioid carcinomas in three age groups (< 50, 50–59, and ≥ 60 years) (Fig. 2). Positive AAPCs for endometrioid carcinomas were significant in the < 50, 50–59, and ≥ 60 years age groups, where the AAPCs were 7.1% (95% CI 6.6–7.6), 6.0% (95% CI 5.1–6.8), and 6.7% (95% CI 6.0–7.5), respectively (Fig. 2A). Similarly, positive AAPCs for nonendometrioid carcinomas were 3.4% (95% CI 1.3–5.5), 5.8% (95% CI 4.0–7.5) and 7.8% (95% CI 6.5–9.2), respectively (Fig. 2B).

### The age-specific incidences of endometrioid and nonendometrioid carcinomas were the steepest in young birth cohorts

**Figure 3:**
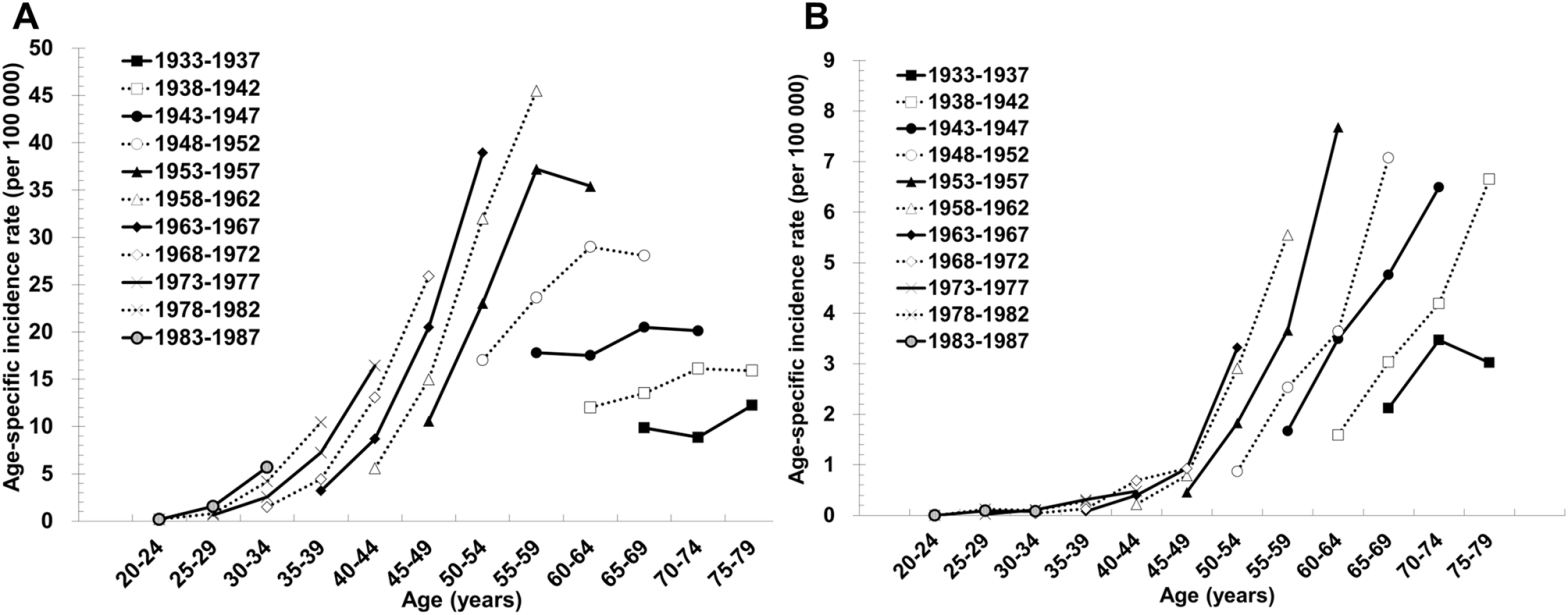
Birth cohort effects on incidence trends of endometrial cancer over time. Age-specific incidence of (**A**) endometrioid carcinoma and (**B**) nonendometrioid carcinoma, grouped by birth cohort.

The trends of age-specific incidences of uterine endometrioid carcinoma, grouped by birth cohort, are shown in Fig. 3A. The age-specific rates began to rise among women born after 1950. The age-specific rates increased rapidly for cohorts born in the 1960s, and the later birth cohorts showed parallel slopes over time. The age-specific incidences of nonendometrioid carcinoma showed trends similar to those observed for endometrioid carcinoma. The age-specific incidences of nonendometrioid carcinoma rose among women born after 1940. The slopes of the incidence curves were similar in the later birth cohorts (Fig. 3B).

### Different stage distributions for endometrioid and nonendometrioid carcinoma

**Figure 4:**
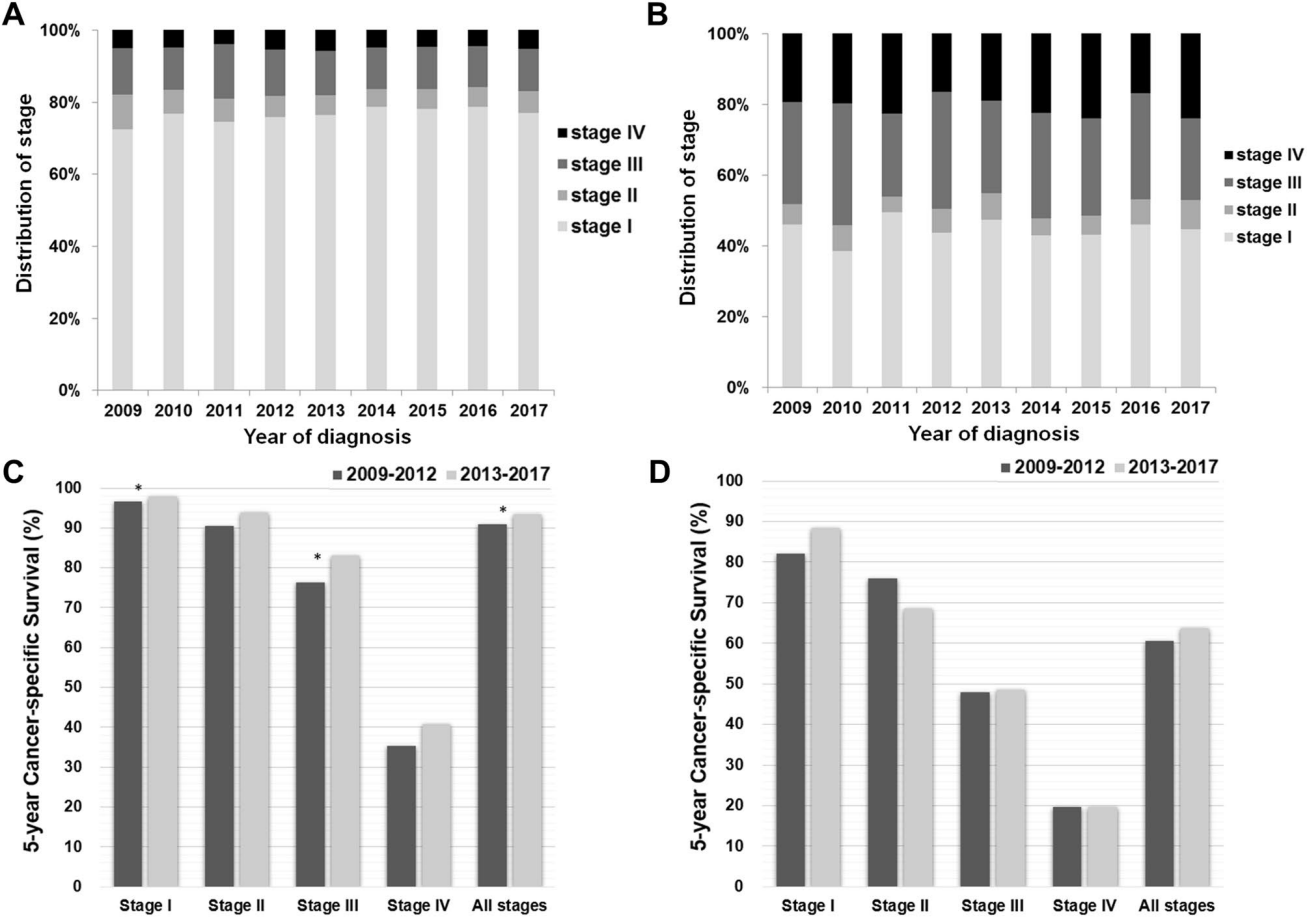
Survival and stage at diagnosis of patients with endometrial cancer in Taiwan. The stages at diagnosis of patients with (**A**) endometrioid and (**B**) nonendometrioid carcinoma between 2009 and 2017. Comparison of 5-year cancer-specific survival rates for the two periods for patients with (**C**) endometrioid and (**D**) nonendometrioid carcinomas by stage. *P < 0.05.

The stage distributions were similar among groups with different years of diagnosis, regardless of histologic type. Early-stage disease (stages I/II) accounted for 80–85% of endometrioid carcinomas in all years of diagnosis (Fig. 4A). In contrast, early-stage disease (stages I/II) accounted for only 50% of nonendometrioid carcinomas. Approximately 30% and 20% of patients presented with stages III and IV disease, respectively (Fig. 4B).

### Factors associated with survival in endometrioid and nonendometrioid carcinoma

A significant difference in the 5-year CSS rates for endometrioid carcinoma was observed between the two periods (Fig. 4C). The CSS rates of stage I patients improved from 96.7 to 97.9% (HR 0.63, 95% CI 0.49–0.81, *P* = 0.0004). The CSS rates of stage III patients also improved from 76.3 to 83.1% (HR 0.72, 95% CI 0.58–0.90, *P* = 0.0039). The 5-year CSS rates of nonendometrioid carcinoma in each stage were lower than those of endometrioid carcinoma (Fig. 4D). We did not observe any significant difference in the CSS rates of all stages for nonendometrioid carcinoma patients in the two periods (60.7% vs. 63.8%, HR 0.92, 95% CI 0.78–1.08, *P* = 0.31). In addition, we analyzed the influences of clinical factors on the 5-year CSS rates in a multivariable analysis (Table 2). After adjusting for other variables, older age at diagnosis was associated with a shorter CSS (HR 1.34, 95% CI 1.03–1.77 for ages 50–59; HR 1.79, 95% CI 1.37–2.38 for ages 60–69; and HR 2.67, 95% CI 2.03–3.57 for ages 70 and older). Women diagnosed in 2013–2017 had significantly higher CSS rates than those diagnosed in 2009–2012 (HR 0.79, 95% CI 0.71–0.87). The CSS rates were lower among patients with stage IV disease (HR 29.55, 95% CI 25.74–33.9) and nonendometrioid carcinoma (HR 2.39, 95% CI 2.14–2.66) than among those with stage I disease and those with endometrioid carcinoma. The risk of nonendometrioid carcinoma was reduced (univariable: HR 6.18, multivariable: HR 2. 39) after adjusting for age, diagnostic period and stage. Stage was the main determinant of CSS in nonendometrioid carcinoma.

**Table 2:** Univariable and multivariable analysis of 5-year cancer-specific survival (CSS) rates for endometrial cancer patients in Taiwan, 2009–2017.

Characteristics	*n*	5-year CSS rate (%)	Univariable			Multivariable		
			HR	95% CI	*P*	HR	95% CI	*P*
**Age (years)**								
< 40	1304	95.3	1.00	Ref		1.00	Ref	
40–49	3145	94.8	1.10	0.82–1.50	0.537	0.84	0.63–1.15	0.264
50–59	6475	90.2	2.12	1.63–2.80	< 0.0001	1.34	1.03–1.77	0.036
60–69	3423	85.3	3.21	2.47–4.26	< 0.0001	1.79	1.37–2.38	< 0.0001
> 70	1524	75.6	5.77	4.40–7.70	< 0.0001	2.67	2.03–3.57	< 0.0001
**Diagnostic period**								
2009–2012	5672	88.0	1.00	Ref		1.00	Ref	
2013–2017	10,199	90.1	0.83	0.75–0.92	0.0004	0.79	0.71–0.87	< 0.0001
**Stage**								
I	11,678	96.6	1.00	Ref		1.00	Ref	
II	963	89.9	3.18	2.50–4.00	< 0.0001	2.94	2.31–3.70	< 0.0001
III	2207	73.2	9.51	8.31–10.89	< 0.0001	7.95	6.93–9.13	< 0.0001
IV	1023	32.4	40.57	35.54–46.4	< 0.0001	29.55	25.74–33.9	< 0.0001
**Histologic type**								
Endometrioid	14,063	92.5	1.00	Ref		1.00	Ref	
Nonendometrioid	1808	62.4	6.18	5.58–6.84	< 0.0001	2.39	2.14–2.66	< 0.0001

*HR* hazard ratio, *CI* confidence interval.

## Discussion

The present cohort study showed that the age-adjusted incidence rates of uterine cancer increased swiftly from 5.3 to 15.2 per 100,000 women between 1998 and 2017. Endometrioid carcinoma accounted for the majority of uterine cancers, and thus, its incidence rates reflected those of all uterine cancers. However, the incidence rates of nonendometrioid carcinoma and uterine sarcoma also increased during the 20-year period. The age-specific incidence of endometrioid carcinoma peaked at 55–60 years of age. In contrast, the incidence rates of nonendometrioid carcinoma continued to increase with age and then plateaued after 60 years of age. Meanwhile, the percentage changes in the incidence of nonendometrioid carcinoma continued to rise with age, particularly among women over 60 years old.

We found that the incidence of endometrioid carcinoma has increased rapidly over the past 20 years, and the trend has continued without reaching a plateau to date. This fast-rising trend was not observed in other Western or Asian countries^22,23^. Trends in the incidence of uterine cancer have also varied with histologic type in other populations^16,24^. Endometrioid carcinoma is estrogen dependent, and obesity is a key driver of temporal trends in its incidence. The prevalence of overweight and obesity is increasing worldwide, but the rates vary by country^25,26^. This variation suggested that different risk profiles and protective factors might be found in different countries^27^. The Nutrition and Health survey conducted in Taiwan (NAHSIT) showed that the location of residence, educational level, and physical activity were correlated with obesity^11^. Three waves of NAHSIT data analyses showed that the percentages of individuals classified as overweight increased from 1993–1996 to 2005–2008 but stabilized between 2005–2008 and 2013. In contrast, over the same time periods, sharp increases were observed in the prevalence of both obesity (11.8%, 17.9%, and 22.1%, respectively) and morbid obesity (0.4%, 0.6%, and 1.4%, respectively) in our country^28^. Rubeis et al. and Song et al. found that overweight in early adulthood was associated with an increased lifetime risk of cancers, including uterine cancer^29,30^. The prevalence of overweight and obesity also increased among children and adolescents in Taiwan^12^. Moreover, the increasing trends in the prevalence of uterine cancer over the study period were more noticeable among younger women than among older women. Previous studies also reported that the most recent birth cohorts showed the greatest increases in the incidences of uterine cancer, particularly endometrioid carcinoma^31,32^, which was similar to our findings.

The measurement methods and classifications of obesity have varied widely. The prevalence of obesity among women was 19.3% in our country during 2013–2016, when obesity was defined as a body mass index (BMI) ≥ 27 kg/m^2^^14^. However, when obesity was defined in terms of the percentage of body fat (≥ 30% body fat for women), the prevalence of obesity increased greatly from 40.2 to 80.8% over the past decade (NAHSIT 1993–1996 to NAHSIT 2005–2008)^33,34^. It has been suggested that the percentage and distribution of body fat could be more closely related to obesity-related disorders than BMI in Asian populations^35–37^. Abdominal obesity (visceral adiposity) is of particular concern in Taiwan, as endometrial cancer is closely associated with obesity.

Women under 50 years of age had the largest upward trend, with 7.1% (95% CI 6.6–7.6) per year for the age-adjusted incidence rate of endometrioid carcinoma. Of note, we observed the highest level of increase among young women aged 30–34 (8.5%, 95% CI 6.9–10.0) (Supplementary Fig. 1). In addition to obesity, changes in other hormonal risk factors should be explored. Potential explanations for the increasing incidence of uterine cancer might be the declining birth rates and a change in parity among Taiwanese women over time. In Taiwan, the average age of women at first delivery increased from 28.8 years in 2008 to 30.9 years in 2018^38^. The risk of uterine cancer decreased with increasing parity^39^. However, the association between the timing of delivery (mother’s age at first and last births) and the risk of uterine cancer was inconclusive^40–42^.

We found that the incidence of nonendometrioid carcinoma also increased significantly. Similarly, in the US, the rates of nonendometrioid cancers rose over time, and the most profound increases were observed among African American women^16,43,44^. Serous carcinoma comprises the majority of nonendometrioid carcinomas, which mainly arise from the loss of p53 function during its molecular pathogenesis^15^. Although obesity was the most common causative factor addressed in studies on the increasing incidence of uterine cancer, it remains unknown whether obesity contributed to the increasing incidence of histologic types other than endometrioid carcinoma. Early diagnosis of nonendometrioid carcinoma had a minimal impact on the incidence because the distribution of disease stages among women diagnosed with nonendometrioid carcinoma remained constant in this study. The distributions of disease stages, grouped according to age at diagnosis, were similar between endometrioid and nonendometrioid carcinomas in this study (data not shown). Other factors that might influence the increasing incidence of uterine cancer should be further explored, rather than focusing solely on obesity.

Younger women and women with early-stage uterine endometrioid carcinoma had better survival rates than their counterparts. Stage was still the strongest predictor of survival even after adjusting for age, diagnostic period, and subtype. Women with nonendometrioid carcinomas had lower CSS rates than those with endometrioid carcinomas. However, the two subtypes differed by age and stage at diagnosis, and after adjustment for covariates, the risk of nonendometrioid versus endometrioid carcinoma was reduced. The diagnostic period was also identified as a prognostic factor in this study. Survival improvements could be attributed to more effective and tailored treatments. For example, a potential benefit might have been conferred by the incorporation of adjuvant chemotherapy over the past decade for patients in advanced stages and women in early stages with high-risk features. Survival might have also been improved by changes in the management of uterine cancer by specialists, such as gynecologic oncologists, in Taiwan and other countries^45^.

The main strength of our study is in the nationally comprehensive analysis of trends in uterine cancer incidence and survival by histologic types. Moreover, we provided age-specific and birth cohort patterns over a 20-year span. To avoid potential bias due to incomplete cancer registry data prior to the enactment of the Cancer Control Act, we compared age-adjusted incidence rates for uterine cancer and nasopharyngeal cancer among women over the same period. The findings showed that this potential bias is small (Supplementary Fig. 3). This study also had some limitations. First, it is a retrospective registry-based study. Consequently, we did not have information on factors that could contribute to the development of uterine cancer, such as BMI, hormonal therapy use, and reproductive history, including parity, pregnancy duration, and the timing and number of births. Another limitation worth noting is that the incidence rate for uterine corpus cancer was uncorrected for hysterectomy. Failure to adjust for hysterectomy prevalence might underestimate the disease burden. For example, the AAPC was reduced from 1 to 0.5% among US women after correction according to SEER 18 (2000–2015)^16^. Hysterectomy prevalence rates vary considerably across countries and ethnicities. The hysterectomy rate is lower in Taiwan, which had a rate of approximately 2.68–3.03 hysterectomies per 1000 women from 1996 to 2001^46^. The total number of hysterectomies in Taiwan remained relatively stable after 2010. We expect that there will be a small decline in the AAPC after correction for hysterectomy, but considering the substantially high AAPC (over 6%) in Taiwan, the corrected incidences and growing trends of endometrioid or nonendometrioid carcinoma are still concerning. Our study was also limited by the absence of a central pathology review, and we could only categorize uterine cancers into four groups (endometrioid, nonendometrioid, sarcoma, and others). Finally, we did not assess the prevalence of overweight and obesity or their association with uterine cancer in this study.

## Conclusion

The incidences of uterine cancers increased significantly, irrespective of histologic type. Age-specific incidences continuously increased over time in cohorts born after 1950. The incidence of endometrioid carcinoma consistently increased in successively younger birth cohorts, and substantial changes in trend were observed among women under the age of 50. These findings underscore the need for etiologic research to clarify the causes of these trends. As increases in aggressive nonendometrioid carcinoma are substantial, guidelines on screening initiation should be considered. New strategies can be implemented to stop the obesity epidemic and increase awareness of the risks and symptoms of uterine cancer through educational campaigns to reduce morbidities from this disease.

## Supplementary Information

Supplementary Figure 1.

Supplementary Figure 2.

Supplementary Figure 3.

## Data Availability

The data that support the findings of this study are available from the corresponding author upon request**.**
