# Supplementary material for: Age-specific trend and birth cohort effect on different histologic types of uterine corpus cancers
Source: Sci Rep. 2023 Jan 19;13:1019. doi: 10.1038/s41598-022-21669-4 (PMC9852563; doi:10.1038/s41598-022-21669-4)
Supplement: Supplementary file 2 — Supplementary Figure 2. [file 41598_2022_21669_MOESM2_ESM.pdf]

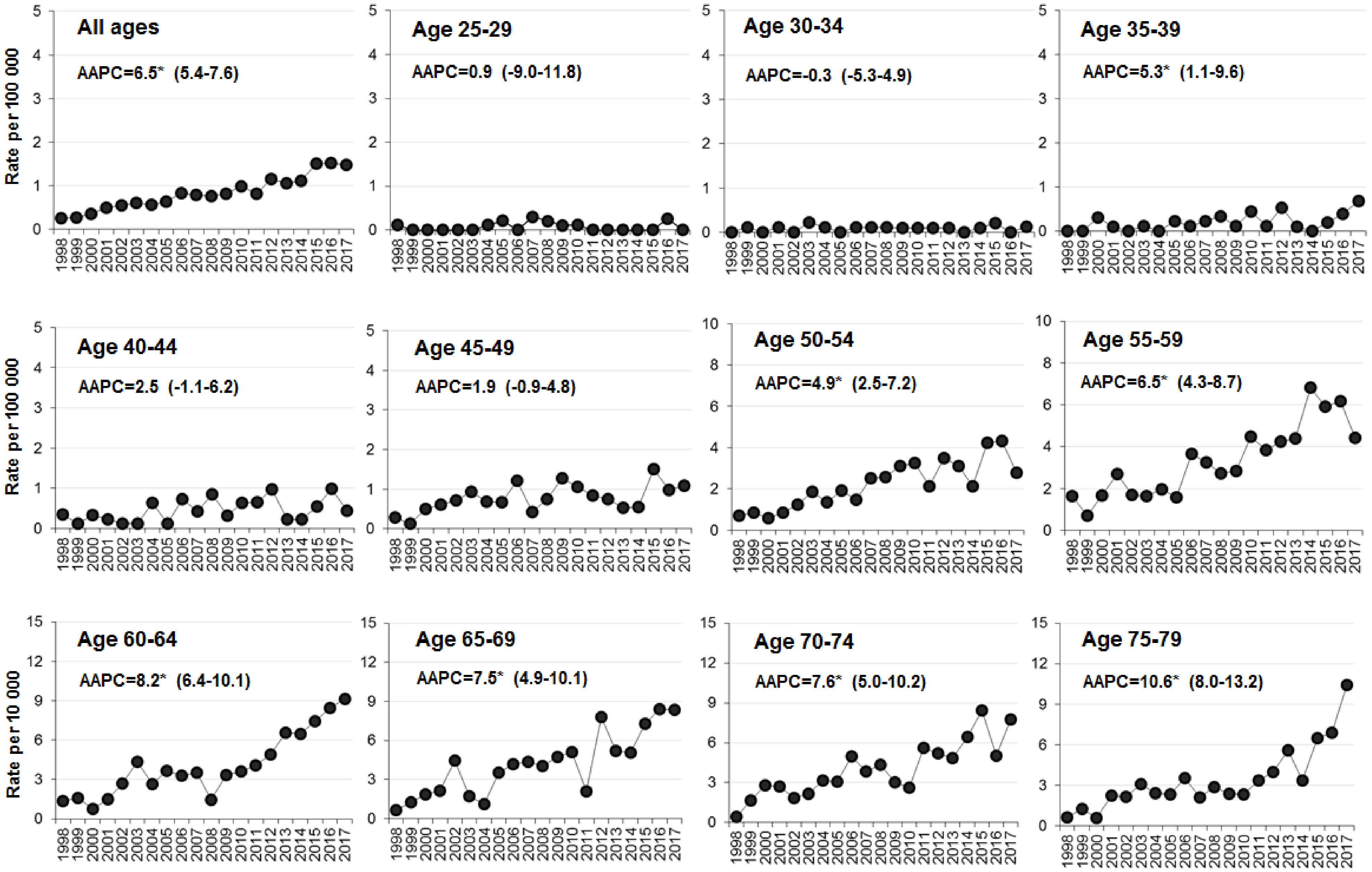

Supplementary Figure 2. Average annual percent changes (AAPCs) and confidence intervals (CIs) of the age-specific incidence rates of uterine nonendometrioid carcinoma in Taiwan from 1998 to 2017.

X-axis: year of diagnosis; Y-axis: age-specific incidence rates. The y-axis scale varies to highlight the temporal trends in incidence.

An asterisk indicates that the AAPC is significantly different from zero (*P*<0.05) using a two-sided test based on the permutation method.
