# Supplementary material for: Age-specific trend and birth cohort effect on different histologic types of uterine corpus cancers
Source: Sci Rep. 2023 Jan 19;13:1019. doi: 10.1038/s41598-022-21669-4 (PMC9852563; doi:10.1038/s41598-022-21669-4)
Supplement: Supplementary file 3 — Supplementary Figure 3. [file 41598_2022_21669_MOESM3_ESM.pdf]

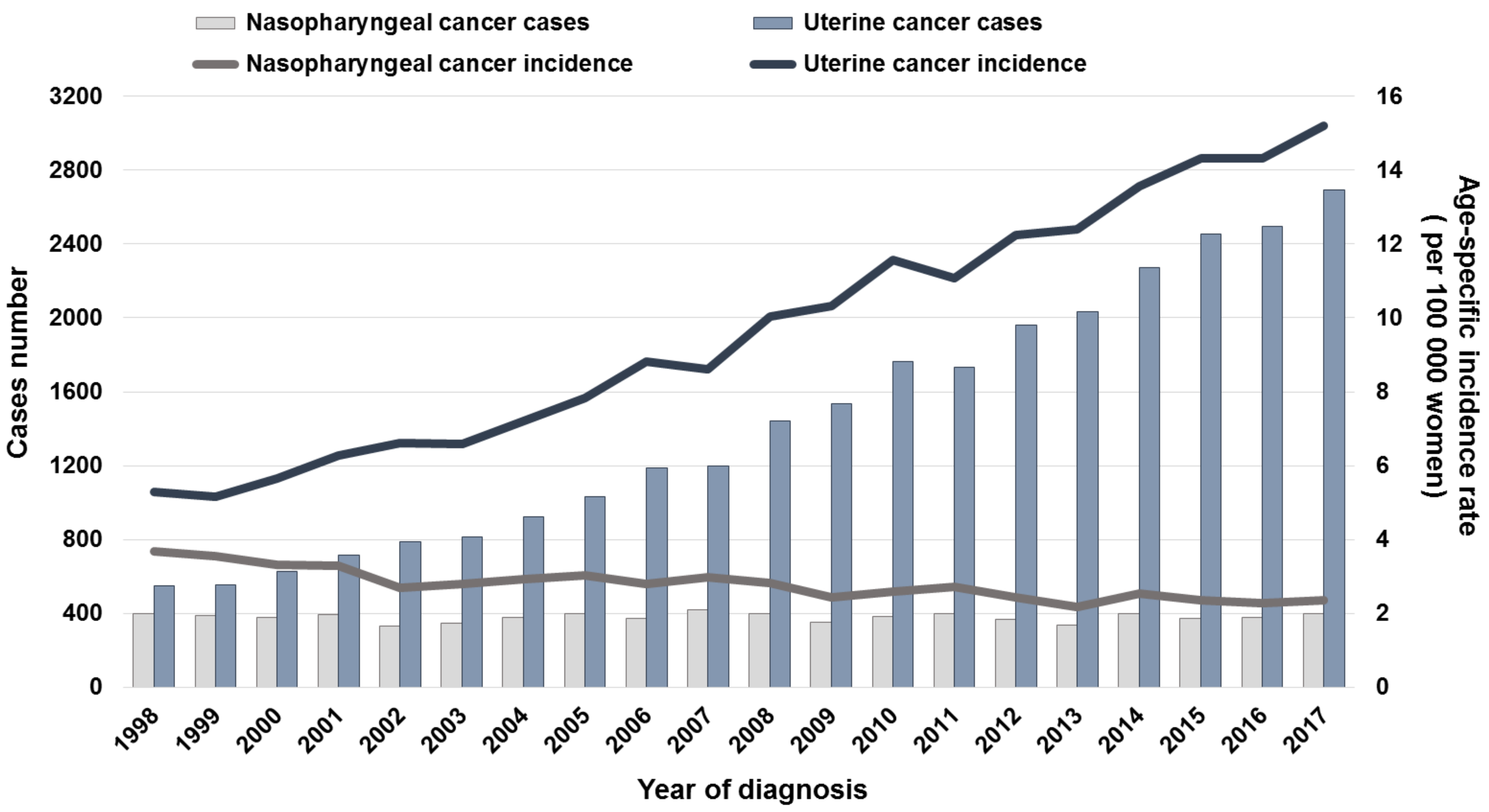

Supplementary Figure 3. The number of cases and trends in age-adjusted incidence rates for uterine corpus cancer and female nasopharyngeal carcinoma in Taiwan from 1998 to 2017.
